# iNOS Promotes the Development of Osteosarcoma via Wnt/*β*-Catenin Pathway

**DOI:** 10.1155/2021/4549221

**Published:** 2021-08-16

**Authors:** Wei Chu, Lirong Cao, Gui Daokun, Jiali Zhao

**Affiliations:** ^1^Department of Orthopedics, The First People's Hospital of Jingzhou (First Affiliated Hospital of Yangtze University), Jingzhou, Hubei, China; ^2^Teaching and Research Division of Surgical Medicine, Hubei College of Chinese Medicine, Jingzhou, China; ^3^Department of Orthopaedics, Lianshui People's Hospital, Lianshui, Jiangsu, China; ^4^Department of Orthopaedics, The Affiliated Huai'an Hospital of Xuzhou Medical University and The Second People's Hospital of Huai'an, Huai'an, Jiangsu, China

## Abstract

Inducible nitric oxide synthase (iNOS), accompanied with protumor and antitumor activity, has been studied in multiple cancers. However, the role of iNOS expression in osteosarcoma (OS) is far from being fully understood. In present work, iNOS levels were detected in OS tissues and cell lines. Colony formation assay, Transwell assay, and fow cytometer were used to assess proliferation, migration, invasion, and apoptosis abilities in vitro after iNOS inhibition. Western blotting determined the expressions of iNOS, MMP2, MMP9, C-MYC, Ki67, PCNA, and *β*-catenin. Mice transfected with OS cells were to evaluate tumor formation. IHC assay was to evaluate the expressions of iNOS and *β*-catenin in mice. The results showed that iNOS was upregulated in both OS tissues and cells compared with that in matched normal tissues or cells. And we found that proliferation, migration, and invasion numbers of OS cells were decreased, and apoptosis numbers of OS cells were increased after iNOS inhibition. MMP2, MMP9, C-MYC, Ki67, and PCNA levels were also reduced in OS cells treated with iNOS inhibition. Else, iNOS inhibition would suppress *β*-catenin expression in OS cells to regulate MMP2, MMP9, C-MYC, Ki67, and PCNA expressions. In addition, tumor formation, iNOS expression, and *β*-catenin expression were inhibited in mice transplanted with iNOS knockout OS cells. These results indicated that iNOS might be a potential therapeutic target for OS.

## 1. Introduction

Osteosarcoma (OS) is the most common primary malignant bone cancer, which occurs frequently in children and adolescents [1]. Epidemiological data reports that there is around 5.2 for children aged 0-19 years per millions of people every year [2]. And the treatment of surgery in combination with chemotherapy increases the survival rate to 60%-75% [3]. However, the existence of highly metastatic potential remains to be major cause of death [4]. Therefore, it is necessary for OC to develop new therapeutic target.

Inducible nitric oxide synthase (iNOS, NOS2) is one kind of nitric oxide synthases that catalyze L-arginine to produce nitric oxide (NO). Its expression is associated with inflammation and malignant diseases [5]. Studies show that iNOS is only nitric oxide synthase (NOS) to participate in tumor progression [6]. And iNOS is highly expressed in tumors such as breast, colon, pancreatic, colon, prostate, ovarian, gastric, bladder, brain, lung cancer, hepatocellular carcinoma, leukemia, head and neck squamous carcinoma, glioblastoma, and melanoma [5–8]. The expression of iNOS is associated with aggressiveness, high histological grade, early recurrences, and poor prognosis of tumor [8–10]. However, the function of iNOS in osteosarcoma development isn't clear yet.

Wnt/*β*-catenin pathway is involved in tumor genesis, proliferation, and chemotherapy resistance [11]. Some reports show that Wnt/*β*-catenin signaling participates in OS development. The Wnt/*β*-catenin pathway is upregulated in OS tissues and cells [12–15]. Wnt/*β*-catenin pathway activates the expression of RUNX2 gene that correlates with metastasis and poor responsiveness to chemotherapy in OS [16]. In OS cells, conventional chemotherapeutics failure is associated with activation of Wnt/*β*-catenin signaling [17]. IWR-1 inhibits OS tumor growth via attenuating Wnt/*β*-catenin pathway [18]. It indicates that Wnt/*β*-catenin signaling can be a therapeutic target for OS metastasis.

Based on above findings, we hypothesize that iNOS plays a regulatory role in the development of OS via Wnt/*β*-catenin pathway and it might be a promising therapeutic target for OS.

## 2. Materials and Methods

### 2.1. Tissue Samples

Forty-five paired tumor tissue samples and matched adjacent normal bone samples were collected from OS patients in The First People's Hospital of Jingzhou. The tissues were quickly stored in liquid nitrogen for subsequent experiments. All patients wrote informed consent. This study was approved by the ethics committee of The First People's Hospital of Jingzhou.

### 2.2. Cell Culture

Human osteosarcoma cell lines (143B and Saos2) and an osteoblast cell line (hFOB1.19) were purchased from the American Type Culture Collection (ATCC) (Rockville, MD). Cells were culture in high-glucose Dulbecco's modified Eagle's medium (DMEM, Gibco, USA) with 10% FBS and 1% antibiotic-antimycotic at 37°C, 5% CO_2_.

### 2.3. Cell Treatment

Cells were seeded on 6-well plates and cultured at 37°C, 5% CO_2_ for 24 h. Then, cells in each well were treated with 2 mL serum-free medium for 1 h before transfection. Empty vector shRNAs and GIPZ lentiviral iNOS (Thermo Fisher Scientific) were transfected into 143B and Saos2 cells via Lipofectamine RNAiMAX (Invitrogen, USA). Another treatment was that cells were treated 1400 W (4 mM) for 96 hours.

### 2.4. Quantitative Real-Time PCR

A TRIzol kit (Invitrogen, Carlsbad, USA) was applied to separate total RNA from the samples or cell lines. The expression of mRNA was measured by SYBR Green (TaKaRa) with the Applied Biosystems 7900 system in accordance with the manufacturer's instructions. The qRT-PCR data were normalized using the 2^−ΔΔ^Ct method. The expression of mRNA was normalized to GAPDH.

### 2.5. Colony Formation

The proliferation ability of 143B and Saos2 cells was detected by clone formation assay. The cells were incubated in six-well plates at 37°C in 5% CO_2_ for 2 weeks. Then, the cells were fixed with 4% paraformaldehyde and stained them with 0.1% Ritz-jimsa dye. The clones were imaged and counted.

### 2.6. BrdU Assay

BrdU assay was applied to detect cell proliferation. Firstly, OS cells were incubated with BrdU for 6 hours and then were incubated with a fluorescein isothiocyanate- (FITC-) labelled antibody (Abcam) against BrdU for 30 minutes. Finally, stained cells were analyzed through a fluorescence microplate reader (Millipore, USA) under the wavelength of 450 nm.

### 2.7. Transwell Assay

The migration and invasion ability of 143B and Saos2 cells was determined by Transwell assay. Cells were cultivated in the top chamber with serum-free DMEM. DMEM with FBS was added to the lower chamber. After 48 hours at 37°C, cells were fixed with 4% paraformaldehyde for 10 minutes and stained with 0.5% crystal viole for 10 minutes at room temperature. Then, cells were counted in three randomly selected microscopic views.

### 2.8. Apoptosis Detection

OS Cells were double-stained with Annexin V-FITC and PI for 35 minutes at room temperature, respectively. Then, a fow cytometer (FCM, Thermo Fisher Scientific, MA, USA) was used to measure cell apoptosis.

### 2.9. Western Blotting

According to the manufacturer's recommendations, protein from cells or tissues was extracted using RIPA buffer (Nacalai Tesque). Equal amounts of protein were separated by SDS–acrylamide gel and transferred into a PVDF membrane (Millipore). After blocking, the membrane was incubated with the following primary human monoclonal antibodies: anti-GAPDH, anti-iNOS, anti-MMP2, anti-MMP9, anti-C-MYC, anti-Ki67, anti-PCNA, and anti-*β*-catenin. Then, the blots were washed with TBS-Tween 20 buffer and incubated with the horseradish peroxidase-conjugated secondary antibodies at room temperature for 1 hour. The signal was visualized by enhanced chemiluminescence (ECL).

### 2.10. Ectopic Tumor Growth

The four-week-old male BALB/c nude mice were purchased from Shimizu Laboratory Supplies (Kyoto, Japan). 143B and Saos2 cells transfected with sh-iNOS were injected into the backs of nude mice. All mice were sacrificed 20 days after coculture of the tumor cells; then, tumor weights were measured. Tumor volumes were calculated by using the formula: volume = 0.4 × width^2^ × length.

### 2.11. Immunohistochemistry (IHC) Assay

The mice tumor tissues were collected and spliced into 5 *μ*m thickness. Then, the expressions of iNOS and *β*-catenin were determined by IHC assay.

### 2.12. Statistical Analysis

Results were represented as mean ± sd. Significance values were compared using Student's *t*-test using GraphPad Prism. *p* < 0.05 was considered statistically significant. All experiments were done in triplicate.

## 3. Results

### 3.1. The iNOS Is Overexpressed in OS

**Figure 1:**
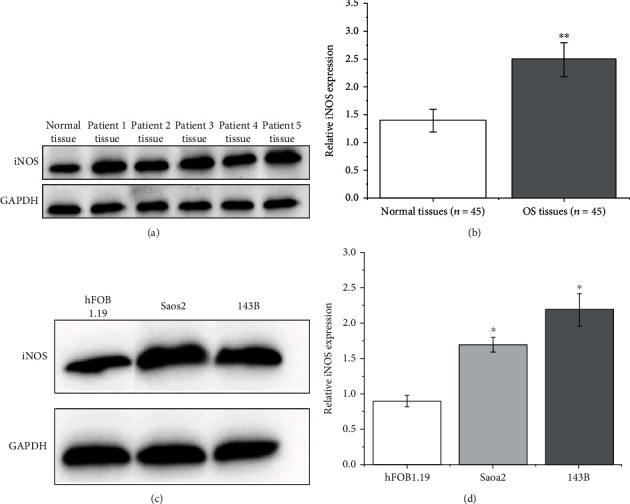
iNOS is upregulated in osteosarcoma (OS) tissues and cells. (a) The representative image of the iNOS expressions in normal tissue and 5 patients' tissues by western blotting. (b) The iNOS expressions in 45 normal tissues and 45 OS tissues (^∗∗^*p* < 0.01). (c, d) The iNOS expressions in hFOB1.19, Saos2, and 143B cells (^∗^*p* < 0.05).

To explore the role of iNOS in the development of OS, we firstly detected the iNOS expression of 45 OS tissues and 45 normal tissues. It was found that iNOS levels in OS tissues were significantly higher than those in normal tissues, as shown in Figures 1(a) and 1(b). Additionally, iNOS in human osteosarcoma cell lines Saos2 and 143B was significantly upregulated than that in osteoblast cell line hFOB1.19, as shown in Figures 1(c) and 1(d).

### 3.2. iNOS Knockdown Inhibits Cell Proliferation, Migration, and Invasion and Promotes Cell Apoptosis in OS

**Figure 2:**
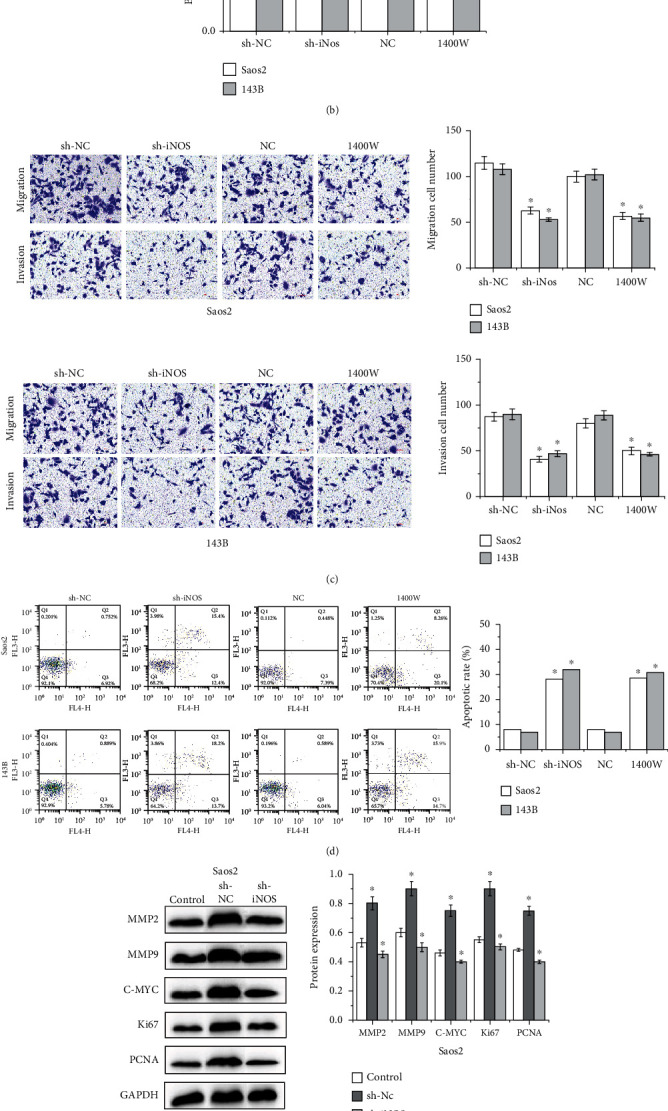
iNOS promotes OS cells proliferation, migration, and invasion and inhibits OS cells apoptosis. (a) The proliferation abilities of Saos2 and 143B cells treated with iNOS inhibition by colony formation assay (^∗∗^*p* < 0.01). (b) The proliferation abilities of Saos2 and 143B cells treated with iNOS inhibition by BrdU assay (^∗^*p* < 0.05). (c) The migration and invasion abilities of Saos2 and 143B cells treated with iNOS inhibition (^∗^*p* < 0.05). (d) The apoptosis abilities of Saos2 and 143B cells treated with iNOS inhibition (^∗^*p* < 0.05). (e, f) The expressions of MMP2, MMP9, C-MYC, Ki67, and PCNA in Saos2 and 143B cells treated with iNOS inhibition.

Then, colony formation assay and BrdU assay were used to testify the function of iNOS on the OS cell proliferation. The results showed that iNOS knockdown or iNOS inhibitor 1400 W suppressed the proliferation of Saos2 and 143B cells, as shown in Figures 2(a) and 2(b). Else, Transwell assay showed that iNOS knockdown or iNOS inhibitor 1400 W suppressed cell migration and invasion of Saos2 and 143B cells, as shown in Figure 2(c). Apoptosis assay showed that iNOS knockdown or iNOS inhibitor 1400 W induced the apoptosis of Saos2 and 143B cells in Figure 2(d). It was also observed that the expressions of MMP2, MMP9, C-MYC, Ki67, and PCNA in Saos2 and 143B cells were higher than those in hFOB1.19 cells. However, Saos2 and 143B cells treated with iNOS knockdown would significantly suppress the expressions of MMP2, MMP9, C-MYC, Ki67, and PCNA in OS cells, as shown in Figures 2(e) and 2(f).

### 3.3. Effect of iNOS on Wnt/*β*-Catenin Pathway of OS Cells

**Figure 3:**
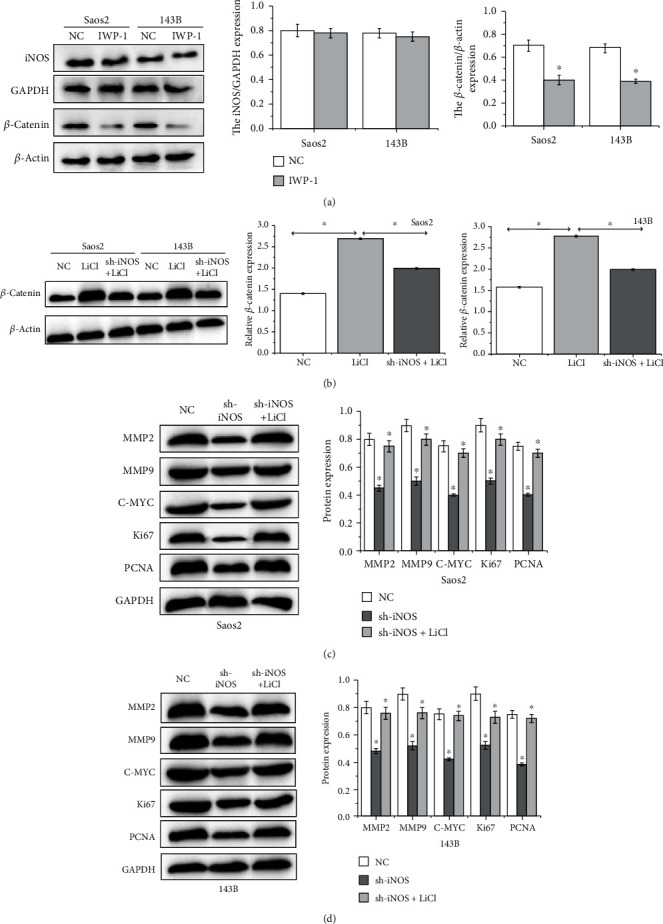
iNOS promotes OS progression via Wnt/*β*-catenin pathway. (a) The iNOS and *β*-catenin expressions in Saos2 and 143B cells treated with Wnt/*β*-catenin pathway inhibitor IWP-1 (^∗^*p* < 0.05). (b) The *β*-catenin expressions in Saos2 and 143B cells treated with NC, Wnt/*β*-catenin pathway activator LiCl, and sh-iNOS+LiCl (^∗^*p* < 0.05). (c, d) The expressions of MMP2, MMP9, C-MYC, Ki67, and PCNA in Saos2 and 143B cells treated with NC, sh-iNOS, and sh-iNOS+LiCl (^∗^*p* < 0.05).

Subsequently, the signaling pathway of iNOS acting on the OS progression was explored. Firstly, we demonstrated that the expression of *β*-catenin was significantly reduced in OS cells treated with the inhibitor of Wnt/*β*-catenin pathway IWP-1, but the expression of iNOS was not significantly changed in Figure 3(a). Then, we found that the expression of *β*-catenin was significantly increased in OS cells treated with the activator of Wnt/*β*-catenin pathway, and the extra sh-iNOS supplement under the basis of which would decrease the expression of *β*-catenin in OS cells in Figure 3(b). It could show that Wnt signaling was not parallel pathway but downstream target of iNOS. Furthermore, it was found that MMP2, MMP9, C-MYC, Ki67, and PCNA expressions in OS cells supplied with Wnt production activator LiCl were evidently increased than those in untreated OS cells. However, extra iNOS inhibition in OS cells treated with LiCl would decrease MMP2, MMP9, C-MYC, Ki67, and PCNA expressions than those in OS cells supplied with LiCl alone, as shown in Figures 3(c) and 3(d).

### 3.4. Effect of iNOS on Tumorigenicity of OS Cells In Vivo

**Figure 4:**
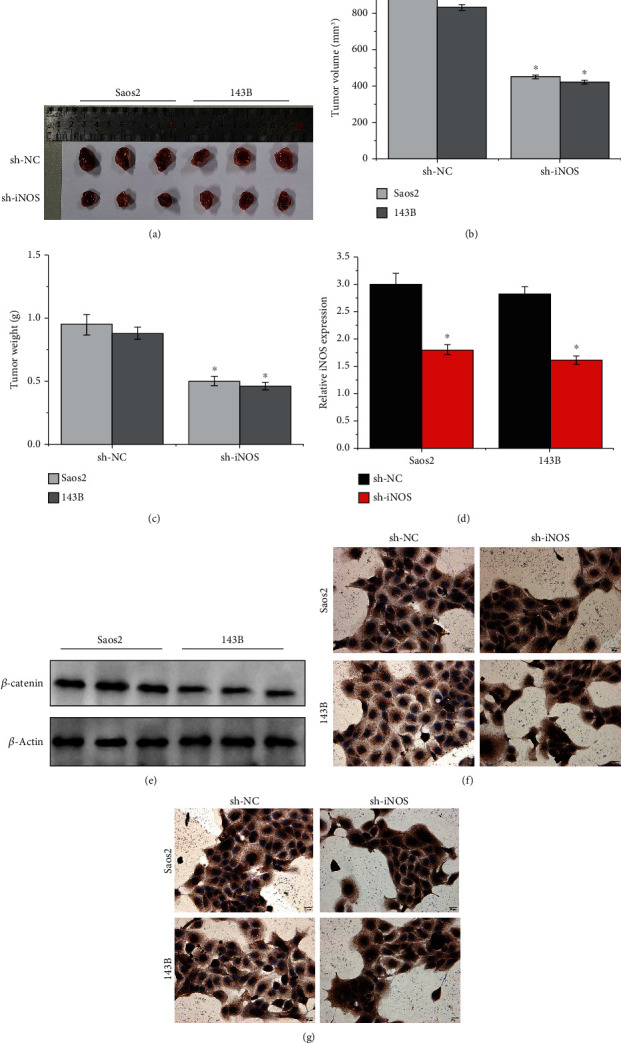
iNOS promotes OS tumor growth. (a) Tumorigenesis in nude mice inoculated with sh-NC and sh-iNOS. (b) The tumor volume change in nude mice (^∗^*p* < 0.05). (c) The tumor weight change in nude mice (^∗^*p* < 0.05). (d) The relative iNOS expression in tumor in mice transplanted with sh-NC and sh-iNOS (^∗^*p* < 0.05). (e) The *β*-catenin expressions in nude mice transplanted with OS cells treated with sh-iNOS. (f) IHC assay detected the iNOS in tissues collected from OS mice. (g) IHC assay detected the *β*-catenin in tissues collected from OS mice.

Mice were supplied with OS cells treated with sh-iNOS or sh-NC, and in vivo experiments showed that iNOS inhibition would suppress tumor growth in OS, as shown in Figure 4(a). The tumor volume and tumor weight were significantly reduced after iNOS knockdown, as shown in Figures 4(b) and 4(c). Also, it showed that iNOS levels were downregulated in tumor treated with sh-iNOS than those in tumor treated with sh-NC, as shown in Figure 4(d). Western blotting showed that the expression of *β*-catenin was decreased in OS tumor tissues treated with sh-iNOS in Figure 4(e). IHC assay also proved that the expressions of iNOS and *β*-catenin were reduced in OS tumor tissues transplanted with sh-iNOS than those in OS tumor tissues transplanted with sh-NC.

## 4. Discussion

The studies of iNOS function in OS pathogenesis are still limited. Therefore, we detected mRNA and protein expression of iNOS in tumor tissues and matched adjacent tissues. We found that iNOS level was significantly higher in OS tissues or cells than that in normal tissues or cells. These findings are in agreement with previous studies in tumors. Granados-Principal et al. found that iNOS was highly expressed in triple-negative breast cancer [8]. Benkhelifa et al. observed that significantly increased iNOS mRNA expression in metastatic tissues of colorectal cancer compared to primary tumors and unaffected mucosa [19]. Pereira et al. showed that iNOS expressions were significantly increased in cancer-associated fibroblasts in pancreatic ductal adenocarcinoma [20].

Further studies showed that the downregulation of iNOS inhibited proliferation and metastasis of OS cells and inhibited apoptosis of OS cells. In human intrahepatic cholangiocarcinoma, iNOS knockdown and iNOS inhibitor (1400 W) suppressed cell proliferation, invasion, and migration [21]. iNOS system played a dominant role in increasing proliferation, migration, and invasion of glioblastoma cells surviving a photochallenge [22]. And we noticed that the function of iNOS in OS was relative with the Wnt/*β*-catenin pathway. Previous studies reported that NOS activity modulates *β*-catenin posttranslational modifications to promote endothelial cell survival [23]. NO acted a mediator in colorectal cancer via Wnt/*β*-catenin pathway, which was closely associated with cancer initiation and metastasis [24]. All findings indicate that iNOS might be an important regulator in OS development.

This work makes a preliminary exploration for the role of iNOS in OS. However, there are some limitations here. The study size in this research is small. And whether iNOS regulates *β*-catenin through nitric oxide is unclear. Else, the effects of iNOS inhibitor can be applied to clinical treatment for OS patients' needs to explore.

In summary, our data demonstrates that iNOS may be a useful biomarker to assess tumor progression for OS. iNOS inhibition plays a negative role in tumor proliferation, migration, and invasion but plays a positive role in tumor apoptosis. And the mechanism underlying iNOS-induced proliferation, migration, invasion, and apoptosis is linked with Wnt/*β*-catenin signaling. Our study provides evidence showing that iNOS may be a potential target for the treatment of OS.

## Data Availability

All data are available upon request.
